# Chicago Dental Society

**Published:** 1890-06

**Authors:** T. L. Gilmer

**Affiliations:** Cor. Sec.


### Chicago Dental Society.

At the annual meeting of the Chicago Dental Society, held on Tuesday, April 1, 1890, the following officers were elected for the ensuing year: President, C. N. Johnson; First Vice-President, C. H. Thayer; Second Vice-President, J. A. Freeman; Secretary, A. E. Baldwin; Corresponding Secretary, T. L. Gilmer; Treasurer, E. D. Swain; librarian, A. W. Harlan; Geo. H. Cushing to succeed himself on the Executive Committee; C. F. Hartt, E. A. Royce and S. B. Palmer, Board of Censors.

T. L. Gilmer, Cor. Sec.

A Progressive Science.—Old Mrs. Bentley: "What a lot of new diseases they have now they didn't have twenty years ago!"

Old Mr. Bentley: "Yes, but you should remember, Eliza, we have a terrible sight more doctors now than we had twenty years ago."—*Judge.*

